# Reconstruction-aware urban crime analytics from incomplete judicial records using heterogeneous graph temporal imputation

**DOI:** 10.3389/fdata.2026.1876811

**Published:** 2026-09-07

**Authors:** Wen Xu, Yong Dai, Qi Zhang, Keyu Chen, Sanxia Zeng

**Affiliations:** 1 Sichuan Police College, Luzhou, Sichuan, China; 2 Intelligent Policing Key Laboratory of Sichuan Province, Luzhou, Sichuan, China; 3 Suining Traffic Police Detachment, Suining Public Security Bureau, Suining, China

**Keywords:** crime data analytics, drug crime classification, explainable artificial intelligence, heterogeneous graph learning, judicial records, missing data reconstruction, public safety intelligence, temporal autoencoder

## Abstract

**Introduction:**

Incomplete judicial records constrain reliable crime analytics because missing attributes, heterogeneous case descriptions, mixed variable types, and imbalanced charge distributions reduce their analytical value. This study developed a reconstruction-aware framework for drug-crime charge classification from incomplete judicial records.

**Methods:**

Drug-related judicial documents from Chengdu, China, covering 2014-2021 were screened, cleaned, and converted into structured person-level observations. The final dataset comprised 12,620 valid documents and 15,184 observations with 16 structured features. A Heterogeneous Graph Convolution Temporal Autoencoder (HetGConv-TAE) was developed to jointly model heterogeneous case-attribute relations and temporal changes in case composition. Performance was evaluated under 10%, 20%, and 30% controlled missingness, followed by XGBoost charge classification and SHAP interpretation.

**Results:**

HetGConv-TAE achieved the best mean reconstruction performance across conventional, static graph, temporal, and relational graph baselines. XGBoost trained on HetGConv-TAE reconstructed data achieved weighted F1 scores close to 80% and ROC-AUC values above 90%. SHAP analysis identified drug weight, place category, and administrative district as the most influential predictors across charge categories.

**Discussion:**

Combining label-excluded heterogeneous graph reconstruction, temporal encoding, downstream classification, and interpretable analysis improves the analytical usefulness of incomplete judicial archives. The framework is intended to support data-quality assessment and aggregate public-safety research rather than automated legal decision-making.

## 1 Introduction

Urban public safety is a fundamental component of sustainable and resilient city governance. Crime affects neighborhood security, social trust, public space quality, and the capacity of local governments to provide stable public services. Drug-related crime is particularly important in this context because it is embedded not only in legal enforcement systems but also in urban spatial organization, population mobility, social activity patterns, and local governance practices. Recent global evidence suggests that drug markets have become larger and more complex, with synthetic substances, online transaction channels, and fragmented trafficking networks reshaping the organization and concealment of drug activities (United Nations Office on Drugs and Crime, 2025). These changes increase the need for data driven analytical tools that can support crime pattern understanding, judicial information processing, and public safety intelligence (Deng et al., 2023; Zhou et al., 2023).

Judicial records provide an important empirical basis for crime analytics because they contain legally validated information on case facts, offender characteristics, offense circumstances, drug type, drug weight, place category, administrative district, and judicial outcomes. Compared with incident level police records or aggregate crime statistics, judicial documents offer richer descriptions of legally relevant facts and case contexts. In principle, these records can support the analysis of how drug crime is distributed across urban space and how different charge categories are formed in judicial practice. However, the analytical use of judicial records remains constrained by the unstructured nature of legal texts, inconsistent documentary completeness, heterogeneous case descriptions, mixed variable types, and strong imbalance across charge categories. These limitations are especially consequential when judicial records are transformed into structured datasets for downstream predictive modeling.

A central challenge in this setting is data incompleteness. Missing values in judicial records may arise from incomplete documentary narratives, differences in case reporting standards, evidentiary limitations, cross system integration problems, and errors introduced during structured coding. In drug crime analytics, missing entries in variables such as drug weight, drug type, offense location, place category, administrative district, prior record, and co-offending status are not merely technical defects. They may directly affect the representation of legally meaningful facts and urban context. If such missingness is handled through simple deletion or routine imputation, the resulting data may distort feature distributions, weaken minority class signals, and reduce the reliability of charge classification. Therefore, incomplete judicial records should be treated as a substantive data quality problem within public safety analytics rather than as a minor preprocessing issue.

Existing studies provide several methodological foundations for the present study. Crime prediction and public safety analytics have developed statistical learning, machine learning, deep learning, and hybrid methods for crime forecasting, hotspot prediction, and risk analysis (Kounadi et al., 2020; Jenga et al., 2023; Du and Ding, 2023; Zhang et al., 2022). Legal NLP studies have improved the structuring and computational analysis of legal documents through legal text representation, information extraction, document classification, and legal decision support (Zhong et al., 2020; Chaitra et al., 2024; Krasadakis et al., 2024). Missing data studies have established the theoretical distinction among missingness mechanisms and developed practical imputation methods, including multiple imputation by chained equations, nearest neighbor imputation, adversarial imputation, and graph neural network-based imputation (Rubin, 1976; van Buuren and Groothuis-Oudshoorn, 2011; Troyanskaya et al., 2001; Yoon et al., 2018; Cini et al., 2022). Heterogeneous graph learning studies have shown that different node types, edge types, and relation structures can be represented through relation-specific aggregation, hierarchical attention, and heterogeneous graph transformers (Schlichtkrull et al., 2018; Wang et al., 2019; Hu et al., 2020). Explainable machine learning further provides tools for interpreting model predictions and assessing feature contributions (Lundberg and Lee, 2017; Dong et al., 2026).

However, these research streams are usually developed separately. Most crime prediction studies focus on downstream prediction and assume that input data are already complete. Legal NLP studies mainly focus on text extraction and document classification, while structured judicial records may still contain missing attributes after extraction. Existing imputation methods are commonly designed for generic tabular data, biomedical data, sensor systems, or multivariate time series rather than judicial records. Heterogeneous graph models are usually used for node classification, link prediction, or representation learning, rather than mixed-type missing data reconstruction in judicial archives. Therefore, incomplete judicial records require a reconstruction-aware framework that jointly considers missing data recovery, heterogeneous legal and urban contextual relations, downstream charge classification, and model interpretation. Table 1 summarizes representative studies and the remaining research gaps.

**Table 1:** Summary of representative studies and research gaps.

Category	Representative studies	Main focus	Gap related to the present study
Crime prediction and public safety analytics	Kounadi et al., 2020; Jenga et al., 2023; Du and Ding, 2023; Zhang et al., 2022	Crime forecasting, hotspot prediction, risk analysis, and interpretable crime prediction	Most studies focus on downstream prediction and usually assume that input data are complete
Legal NLP and judicial document structuring	Zhong et al., 2020; Chaitra et al., 2024; Krasadakis et al., 2024	Legal text representation, information extraction, document classification, and legal decision support	Structured records may still contain missing attributes after legal text extraction
Missing data and imputation	Rubin, 1976; van Buuren and Groothuis-Oudshoorn, 2011; Troyanskaya et al., 2001; Yoon et al., 2018; Cini et al., 2022	Missingness mechanisms, classical imputation, adversarial imputation, and graph neural network based imputation	Existing methods are usually designed for generic tabular data, biomedical data, or sensor time series rather than judicial records
Heterogeneous graph learning	Schlichtkrull et al., 2018; Wang et al., 2019; Hu et al., 2020	Relation specific aggregation, heterogeneous attention, and heterogeneous graph representation	These models are not specifically designed for mixed numerical and categorical missing data reconstruction in judicial archives
Explainable machine learning	Lundberg and Lee, 2017; Zhang et al., 2022	Feature attribution and model interpretation	Explanation methods support interpretation but do not address missing data reconstruction

Missing data reconstruction has also received increasing attention in statistics, machine learning, and data science. Classical approaches such as multiple imputation and K nearest neighbors remain widely used because they are simple and interpretable. More recent methods have introduced adversarial learning, graph neural networks, and temporal representation learning to improve imputation quality in complex datasets (Yoon et al., 2018; Cini et al., 2022; Kim et al., 2025). These methods suggest that reconstruction performance can be improved when models exploit relational structure and temporal dependency rather than treating records as independent rows in a flat table. A comparable trend toward structure-aware and context-sensitive modeling can be observed in recent traffic safety studies using CrossTabNet, dynamic decomposition, and collaborative control frameworks to capture real-time or spatially differentiated risk patterns (Jin et al., 2025, 2026; Ma et al., 2026). Nevertheless, most graph-based imputation studies were developed for sensor systems, infrastructure monitoring, traffic systems, or generic multivariate time series. Judicial records differ from these domains because their relations are semantic, institutional, and urban contextual rather than purely spatial or physical. Case records may be connected through shared drug types, place categories, administrative districts, offender profiles, and temporal conditions. This implies that a reconstruction model for judicial data should be able to represent heterogeneous case attribute relations and temporal changes in case composition.

Legal information extraction and legal artificial intelligence provide another relevant foundation for this study. Recent advances in legal natural language processing have improved the structuring of legal documents, including entity extraction, relation extraction, document classification, and legal decision support (Chaitra et al., 2024; Dehghani et al., 2025; Krasadakis et al., 2024). These developments make it possible to transform large volumes of judicial texts into structured analytical records. However, structured extraction alone cannot eliminate the problem of missing attributes. Even after legal texts are converted into structured variables, the resulting datasets often remain incomplete, heterogeneous, and imbalanced. Therefore, legal document structuring, missing data reconstruction, downstream classification, and model interpretation should be treated as interconnected stages of one analytical workflow rather than as separate technical procedures.

To address these issues, this study develops a reconstruction-aware urban crime analytics framework for drug crime charge classification from incomplete judicial records. Drug-related judicial documents collected in Chengdu, China, from 2014 to 2021 were screened, cleaned, and transformed into structured person-level observations. The final dataset contains 12,620 valid judicial documents and 15,184 person-level observations with 16 structured features covering legal facts, offender characteristics, substance evidence, temporal context, place category, and administrative district. A Heterogeneous Graph Convolution Temporal Autoencoder, termed HetGConv-TAE, is proposed to reconstruct incomplete records by jointly modeling heterogeneous case attribute relations and temporal variation in case composition. The reconstructed datasets are then used for downstream charge classification with XGBoost, and SHAP is applied to interpret the contribution of legally meaningful and urban context related predictors.

**Figure 1:**
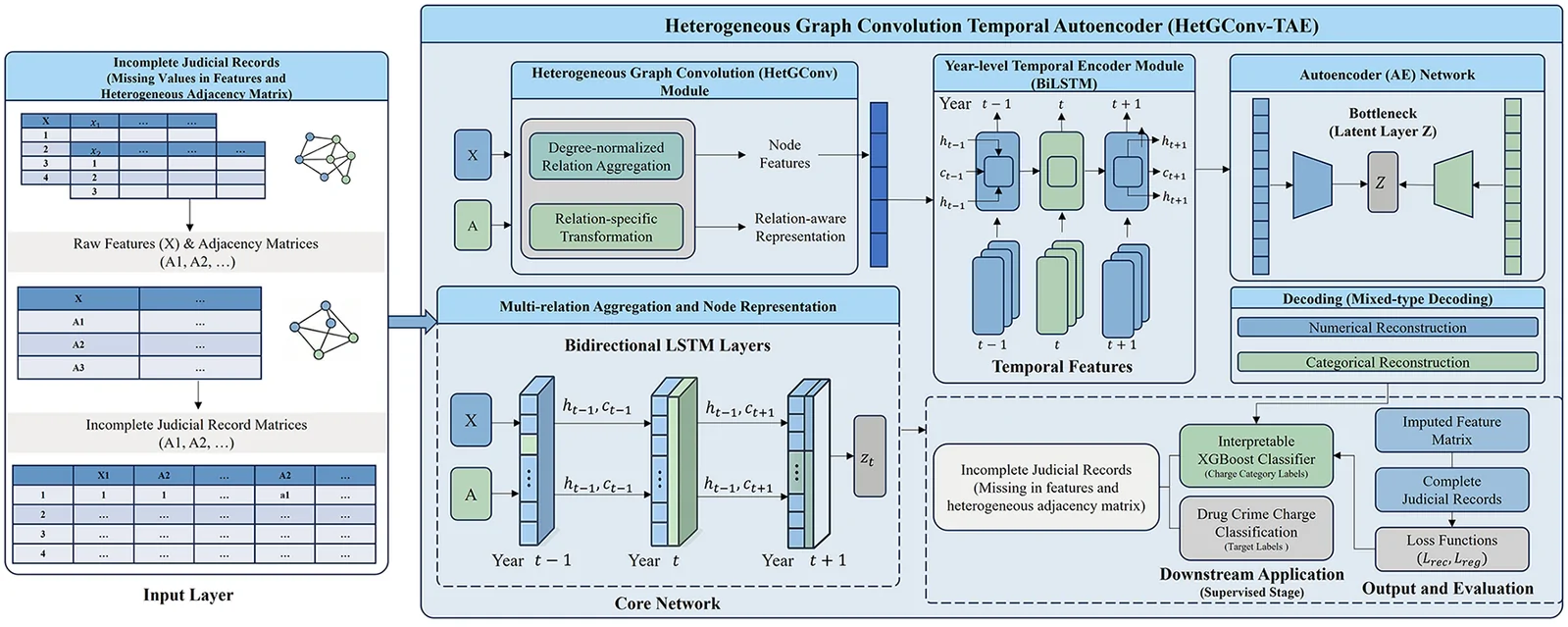
Overall workflow of the proposed label-excluded reconstruction-aware urban crime analytics framework, including judicial document structuring, non-target heterogeneous graph construction, HetGConv-TAE imputation, downstream charge classification, and SHAP-based interpretation.

The overall workflow of the proposed framework is shown in Figure 1. To improve the logical flow of the analysis, the framework is organized as a sequential pipeline with five connected stages. First, drug-related judicial documents are screened, cleaned, and transformed into structured person-level observations. Second, the original missingness pattern is examined, and the structured records are converted into a heterogeneous case attribute graph. Third, HetGConv-TAE reconstructs missing numerical and categorical entries by jointly modeling heterogeneous attribute relations and temporal variation in case composition. Fourth, the completed datasets are used for downstream charge classification with XGBoost to evaluate whether reconstruction improves task performance. Fifth, SHAP is used to interpret the final classifier and examine whether the reconstructed feature space supports legally meaningful and contextually plausible explanations. This pipeline clarifies the relationship between data structuring, missing data reconstruction, classification, and interpretation.

Within this pipeline, the reconstruction stage is separated from the downstream prediction stage. The heterogeneous graph used by HetGConv-TAE is constructed from non-target case attributes, whereas charge categories are introduced only after imputation for supervised classification and interpretation. This separation defines the leakage-control boundary of the framework and ensures that target-label information is not propagated during unsupervised feature reconstruction.

This study contributes to the literature in three respects. First, it reframes incomplete judicial records as a core data quality problem in crime analytics and public safety intelligence. Instead of treating missing values as a routine preprocessing issue, the study explicitly links missing data reconstruction to downstream charge classification. Second, it develops a reconstruction oriented heterogeneous graph temporal framework for judicial records. Unlike general heterogeneous GNNs that mainly support representation learning, node classification, or link prediction, HetGConv-TAE is designed to recover mixed numerical and categorical missing attributes from semantically connected case records. Third, it provides an interpretable analytical pipeline that connects reconstructed data, classification performance, and feature level explanations. The resulting evidence helps clarify how variables such as drug weight, place category, administrative district, co-offending status, and offender characteristics contribute to charge classification under incomplete data conditions.

The remainder of this paper follows the same analytical sequence. Section 2 first describes the data source and structured feature construction, then explains the missingness formulation, heterogeneous graph representation, HetGConv-TAE reconstruction model, downstream classification design, and SHAP interpretation method. Section 3 reports the empirical results in the same order, including dataset structure, missingness patterns, reconstruction performance, ablation analysis, downstream classification performance, and model interpretation. Section 4 discusses the methodological implications, practical use, ethical boundaries, limitations, and future research directions.

## 2 Materials and methods

### 2.1 Data source and structured feature construction

The empirical data were derived from drug-related criminal judgment documents collected in Chengdu, China, covering the period from 2014 to 2021. Chengdu provides a suitable empirical setting for urban crime analytics because it is a large metropolitan region with heterogeneous administrative districts, diverse urban functional spaces, and complex public safety governance conditions. Drug crime cases in this context are not only legal events but also spatially and socially embedded phenomena. Judicial records therefore provide a useful data source for examining how legal facts, offender characteristics, urban context, and charge categories are jointly represented in structured crime data.

Judicial documents were used as the primary data source because they contain legally validated descriptions of case facts, evidentiary information, offender attributes, offense circumstances, and judicial outcomes. The original corpus was collected through keyword-based retrieval and case relevance screening. Documents were excluded if they were duplicated, unrelated to drug crime, incomplete in substantive legal content, or unsuitable for person-level restructuring. After screening and cleaning, 12,620 valid judicial documents were retained for structured processing.

Because the analytical task focuses on charge classification at the person-level rather than at the document level, the cleaned judicial documents were transformed into person-level observations. This step was necessary because a single judicial document may involve multiple offenders, and each offender may have distinct demographic attributes, prior record information, co-offending status, and case involvement characteristics. After person-level restructuring, the final analytical dataset contained 15,184 valid observations with 16 structured explanatory features.

The judicial documents were converted into structured records through a document structuring and verification process. The extracted and constructed fields included judgment title, court, judgment date, offense time, offense location, place category, administrative district, drug type, total net drug weight, gender, age, ethnicity, educational level, occupation, prior record, co-offending status, and charge category. Charge category was retained exclusively as the downstream response label and was not used as an explanatory variable during graph construction or missing data reconstruction. Because charge category represents the downstream outcome evaluated after reconstruction, it was excluded from the attribute node vocabulary, relation type set, adjacency matrices, node features, relation embeddings, imputation inputs, reconstruction loss, and downstream predictor set.

Several raw fields were transformed or regrouped before modeling. Drug type was categorized into four groups: methamphetamine, heroin, mixed methamphetamine and heroin, and other drugs. Drug weight was grouped into three non-overlapping ranges: < =10 g, >10–50 g, and >50 g. The target variable, charge category was regrouped into four classes: smuggling, trafficking, transporting, or manufacturing drugs; sheltering others to take drugs; illegal possession of drugs; and other charges. This regrouping strategy was adopted to reduce excessive sparsity while preserving legal interpretability and empirical feasibility under imbalanced class distributions. This regrouped charge variable was used to define the downstream multiclass task and to describe class imbalance, but it was not used to create graph nodes or graph edges during the unsupervised reconstruction phase.

Temporal variables were derived from offense time and judgment date, including year, month, day of week, season, holiday status, and pandemic period. Although temporal variables were not treated as the primary determinants of charge classification, they were included to capture changes in urban activity patterns, enforcement conditions, and case composition over time. Spatial and contextual variables included offense location, place category, and administrative district. These variables connect person-level judicial records to the geography of urban crime governance and allow the model to capture contextual heterogeneity across urban spaces. Table 2 presents the core variables used in this study, including judicial identifiers, event time context, event space context, substance evidence, offender profile, case organization, and charge category.

**Table 2:** Core variables extracted from judicial documents and their corresponding data types.

Variable group	Variables	Type	Remarks
Judicial identifiers	Judgment title, judgment court, judgment date	Text/date	Used in record management and extraction verification
Event time context	Offense time, month, day of week, season, holiday, pandemic period	Date/categorical	Capture temporal and contextual variation
Event space context	Offense location, place category, administrative district	Text/categorical	Represent spatial and venue characteristics
Substance evidence	Drug type, total net drug weight	Categorical/ordinal	Legally salient indicators of offense seriousness
Offender profile	Gender, age, ethnicity, education, occupation, prior record	Mixed	Describe demographic and criminal history characteristics
Case organization	Co-offending status	Binary categorical	Distinguishes single offender and multi offender structure
Target label	Charge category	Multiclass	Downstream outcome; excluded from HetGConv-TAE graph construction, imputation inputs, relation types, adjacency matrices, and classifier predictors

Table 3 summarizes the distribution of drug types and charge categories in the final analytical dataset. Methamphetamine related cases formed the dominant drug type, while smuggling, trafficking, transporting, or manufacturing drugs represented the majority charge category. This distribution indicates strong class imbalance, which motivated the use of evaluation metrics beyond accuracy in the downstream classification task.

**Table 3:** Distribution of drug types and charge categories in the final dataset.

Category type	Class	Count	Share (%)
Drug type	Methamphetamine	12,963	85.4
Drug type	Heroin	917	6.0
Drug type	Methamphetamine and heroin	323	2.1
Drug type	Other drugs	853	5.6
Drug type	Missing	128	0.8
Charge	Smuggling, trafficking, transporting, or manufacturing	9,528	62.7
Charge	Sheltering others to take drugs	4,133	27.2
Charge	Illegal possession of drugs	1,360	8.9
Charge	Other charges	163	1.1

Drug-type shares are calculated using all 15,184 observations; percentages may not total 100% because of rounding.

**Figure 2:**
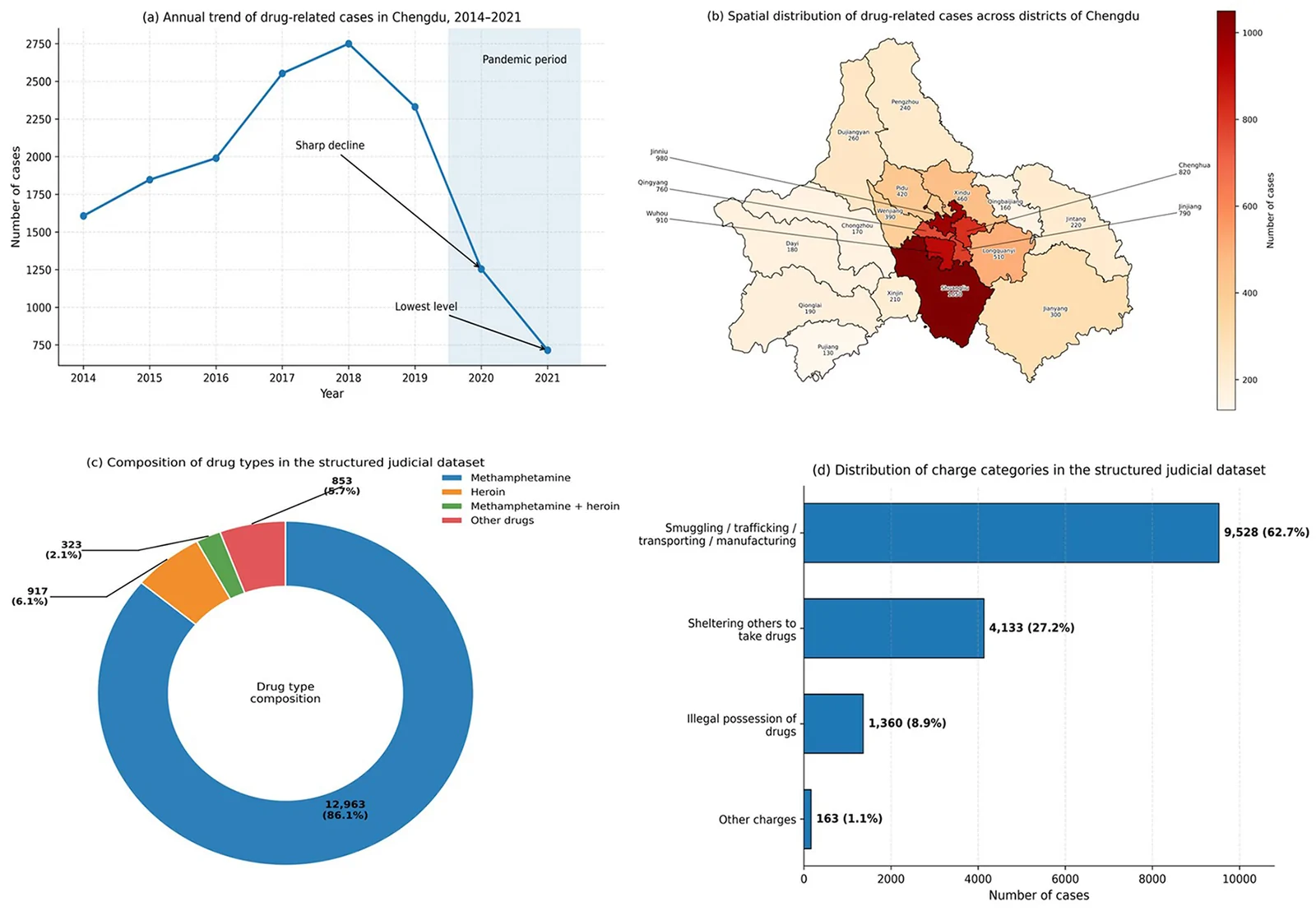
Descriptive overview of the structured judicial dataset. **(a)** Annual trend of drug-related person-level observations in Chengdu from 2014 to 2021. **(b)** Spatial distribution of drug-related person-level observations across districts of Chengdu. **(c)** Composition of drug types among the 15,056 observations with non-missing drug-type information. **(d)** Distribution of charge categories among all 15,184 person-level observations.

Before model construction, descriptive analysis was conducted to examine the empirical structure of the judicial dataset. The annual distribution of cases was used to characterize temporal variation. The administrative district distribution was used to examine spatial heterogeneity across Chengdu. The drug type composition and charge category distribution were used to assess feature imbalance and target imbalance. Figure 2 presents the descriptive overview of the structured judicial dataset, including annual distribution, district level distribution, drug type composition, and charge category distribution.

The descriptive results indicate that the dataset is not a balanced and complete tabular dataset. Instead, it is temporally uneven, spatially heterogeneous, semantically mixed, and imbalanced across legal outcomes. These characteristics provide the empirical basis for the proposed reconstruction-aware modeling framework.

### 2.2 Missingness problem and heterogeneous graph representation

Missingness in judicial records was treated as a substantive data quality problem rather than as a routine preprocessing issue. Missing values may arise from incomplete documentary narratives, differences in writing style across courts and years, evidentiary incompleteness, inconsistent reporting standards, and errors introduced during the transformation of legal texts into structured records. In the context of drug crime analytics, missing entries in variables such as drug weight, drug type, offense location, place category, administrative district, prior record, and co-offending status may affect the representation of legally meaningful facts and urban contextual information.

To examine the original missingness mechanism, this study constructed missingness indicators for variables with incomplete entries and tested whether these indicators were associated with observed temporal, spatial, legal, and offender-related variables. Chi-square tests and Fisher's exact tests were used to examine associations between missingness indicators and observed variables, while Little's MCAR test was used to evaluate whether the overall missingness pattern was completely random. These analyses were used to determine whether the observed missingness pattern was compatible with pure MCAR or whether missingness showed systematic associations with observed temporal, spatial, legal, and offender-related variables. Such associations can provide evidence consistent with MAR or context-dependent missingness, but they cannot by themselves establish MAR or exclude MNAR.

The mathematical formulation of the proposed framework is presented in (1)–(16). Let the structured dataset be denoted as:


$$\begin{array}{l} \text{X}=\left[x_{ij}\right]\in {\mathbb{R}}^{n\times p}, \end{array}$$ (1)

where *n* denotes the number of person-level observations and *p* denotes the number of structured features. Because the dataset contains missing values, a binary observation mask is defined as


$$\begin{array}{l} \text{M}=\left[m_{ij}\right]\in {\left\{0,1\right\}}^{n\times p}, \end{array}$$ (2)

where *m*_*ij*_ = 1 if the value of feature *j* for observation *i* is observed, and *m*_*ij*_ = 0 otherwise. The observed incomplete data matrix can be expressed as:


$$\begin{array}{l} {\text{X}}^{obs}=\text{M}⊙\text{X}, \end{array}$$ (3)

where ⊙ denotes elementwise multiplication. The objective of missing data reconstruction is to estimate a completed data matrix $\hat{\text{X}}$ while preserving observed entries and recovering missing entries. The final completed matrix can be written as


$$\begin{array}{l} {\text{X}}^{comp}=\text{M}⊙{\text{X}}^{obs}+\left(1-\text{M}\right)⊙\hat{\text{X}}. \end{array}$$ (4)

**Figure 3:**
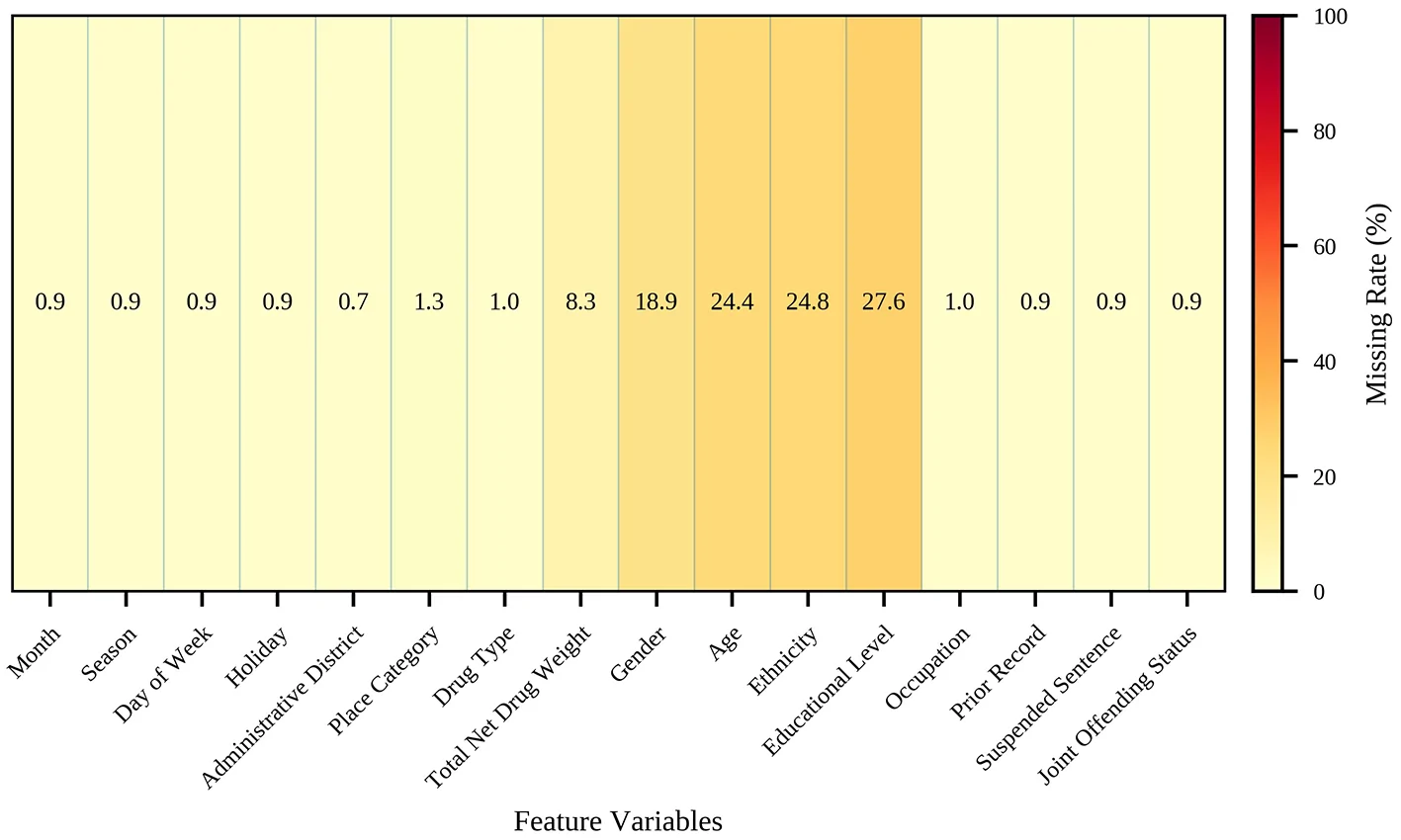
Variable-wise missingness distribution in the structured judicial dataset.

This formulation ensures that the observed values remain unchanged and only missing entries are replaced by reconstructed values. The reconstructed dataset is then used as the input for downstream charge classification. Figure 3 presents the variable-wise missingness distribution of the structured judicial dataset. The missingness pattern is uneven across variables and affects several legally and contextually relevant features. This indicates that missing data are not confined to peripheral attributes. Instead, missingness may influence key predictive signals required for charge classification. Such a pattern justifies the need for a dedicated reconstruction framework rather than simple deletion or routine imputation.

To preserve the semantic relations among judicial records and their associated attributes, the structured tabular data were transformed into a label-excluded heterogeneous case-attribute graph. The graph was constructed from 15,184 person-level observations, each of which was represented as a case node. Attribute nodes were generated from observed non-target structured variables and were grouped into five semantic types: substance evidence nodes, offender profile nodes, urban context nodes, temporal context nodes, and case organization nodes. This design follows a target-leakage prevention principle: charge category was treated as the downstream response variable and was not used to construct attribute nodes, edge types, adjacency matrices, node features, relation embeddings, graph-convolution inputs, or the masked reconstruction loss. Charge labels were retained for missingness mechanism diagnosis and downstream charge classification after imputation, but they were not available to the unsupervised representation-learning stage. Edges were constructed according to observed non-target case-attribute membership. A case node was connected to an attribute node when the corresponding non-target value was observed in the structured record. The main edge types therefore included drug type and drug weight relations, offender profile relations, administrative district relations, place category relations, co-offending relations, and year relations. No charge-category relation was included in the graph used for imputation. After excluding the four charge-category nodes and the corresponding case-charge edges, the label-excluded graph contained 15,184 case nodes, 124 non-target attribute nodes, and 57,699 observed non-target case-attribute edges.


$$\begin{array}{l} G=\left(V,E,R\right), \end{array}$$ (5)

where V denotes the node set, E denotes the edge set, and R denotes the set of relation types. Each edge links a case node to an observed non-target attribute node. For example, a case node may be connected to a district node, a place category node, a drug type node, a drug weight group node, a co-offending status node, and a year node, but not to a charge-category node. Through these edges, the graph represents shared evidentiary, contextual, demographic, organizational, and temporal attributes among cases while keeping the downstream label outside the imputation model.

This graph construction differs from a conventional homogeneous graph because relations among records are not defined only by physical proximity or statistical similarity. Instead, the relations are semantic, legal, and urban contextual. Cases may be similar because they share the same drug type, occur in the same administrative district, involve similar place categories, or belong to the same temporal period. Encoding these relations explicitly allows the reconstruction model to exploit information that is difficult to capture through flat tabular imputation.

### 2.3 HetGConv-TAE reconstruction framework

A Heterogeneous Graph Convolution Temporal Autoencoder, termed HetGConv-TAE, was developed to reconstruct incomplete judicial records. Existing heterogeneous graph neural networks, such as R-GCN, HAN, and HGT, provide general mechanisms for modeling multi relational graphs, heterogeneous information networks, and type dependent graph representations. These models are mainly designed for graph representation learning, node classification, or link prediction. They do not directly target mixed-type missing data reconstruction in judicial archives.

Different from these models, HetGConv-TAE is organized around reconstruction-aware judicial record analytics. As summarized in Table 4, it constructs a case attribute heterogeneous graph from person-level judicial records, combines relation-aware aggregation with year-level temporal autoencoding, reconstructs both numerical and categorical missing entries through a mixed-type masked loss, and evaluates the completed data through downstream charge classification and SHAP-based interpretation. Therefore, the methodological contribution of HetGConv-TAE lies in adapting heterogeneous graph temporal learning to incomplete judicial records and linking reconstruction quality with downstream legal classification performance.

**Table 4:** Comparison between HetGConv-TAE and representative heterogeneous GNNs.

Model	Main design	Relevance to the present study
R-GCN	Uses relation-specific transformations to model multi relational graphs	Provides a basis for relation-aware aggregation, but does not target mixed-type missing data reconstruction in judicial records
HAN	Uses meta path-based attention to learn representations in heterogeneous information networks	Captures heterogeneous semantics, but depends on predefined meta paths and is not designed for judicial record imputation
HGT	Uses type dependent attention to encode heterogeneous graph structures	Supports heterogeneous representation learning, but focuses mainly on graph encoding rather than masked reconstruction and downstream legal classification
HetGConv-TAE	Combines a label-excluded case-attribute graph, relation-normalized aggregation, year-level temporal autoencoding, and mixed-type masked reconstruction	Designed to reconstruct incomplete non-target judicial-record features and validate the completed data through charge classification and SHAP interpretation

The label-excluded graph construction is therefore part of the methodological design rather than a *post hoc* preprocessing detail. It keeps the reconstruction objective focused on observed non-target attributes and reduces the risk that the completed feature matrix is influenced by target-label leakage.

Let ${\text{h}}_{v}^{\left(l\right)}$ denote the representation of node *v* at graph convolution layer *l*. A general relation-aware heterogeneous aggregation process can be written as


$$\begin{array}{l} {\text{h}}_{v}^{\left(l+1\right)}=\sigma \left(\underset{r\in R}{\sum }\underset{u\in N_{r}\left(v\right)}{\sum }\alpha_{uv}^{r}{\text{W}}_{r}^{\left(l\right)}{\text{h}}_{u}^{\left(l\right)}\right), \end{array}$$ (6)

where $N_{r}\left(v\right)$ denotes the set of neighbors of node *v*under relation type *r*, $\alpha_{uv}^{r}$ denotes the relation-specific aggregation weight between node *u*and node *v*, ${\text{W}}_{r}^{\left(l\right)}$ denotes the relation-specific transformation matrix, and σ(·) denotes a non-linear activation function. This formulation allows the model to learn different contribution patterns from different relation types.

For relation-aware aggregation, we used a fixed degree-normalized coefficient rather than a learned attention score to control the contribution of neighboring nodes under each relation type. The coefficient was defined as:


$$\begin{array}{l} \alpha_{uv}^{r}=\frac{1}{\sqrt{d_{u}^{r}d_{v}^{r}}}, \end{array}$$ (7)

where $d_{u}^{r}$ and $d_{v}^{r}$ denote the relation-specific degrees of nodes *u* and *v*, respectively. Relation specific expressiveness was captured by the transformation matrix associated with relation r and the corresponding relation embedding. Charge category nodes and charge derived relations were excluded from this aggregation process.

The temporal slicing strategy followed the annual structure of the judicial records. The heterogeneous graph was divided into eight yearly slices from 2014 to 2021. Because different yearly slices contained different numbers of case nodes, the temporal branch did not require node-wise alignment across years. Instead, the heterogeneous graph convolution module first produced a case-level embedding **h**_*i, t*_ for each case *i* in year *t*.

A masked mean pooling operation was then used to obtain a fixed-dimensional representation for each yearly slice:


$$\begin{array}{l} z_{t}=\frac{1}{\left|V_{t}^{obs}\right|}\underset{i\in V_{t}^{obs}}{\sum }{\text{h}}_{i,t} \end{array}$$ (8)

where $V_{t}^{obs}$ denotes the set of valid case embeddings in year *t*.

The ordered sequence {*Z*_1_, …, *Z*_*T*_} was then processed by a bidirectional LSTM. The forward and backward hidden states were obtained as:


$$\begin{array}{l} {\vec{S}}_{t}=LSTM_{f}\left(Z_{t},{\vec{S}}_{t-1}\right),\;{\overset{⃖}{S}}_{t}=LSTM_{b}\left(Z_{t},{\overset{⃖}{S}}_{t+1}\right), \end{array}$$ (9)

and the year-level temporal context was defined as


$$\begin{array}{l} S_{t}=\left[{\vec{S}}_{t}||{\vec{S}}_{t}\right] \end{array}$$ (10)

To preserve case-level reconstruction, the year-level temporal context was concatenated with the graph embedding of each case belonging to the corresponding year:


$$\begin{array}{l} {\tilde{\text{h}}}_{i,t}=\left[{\text{h}}_{i,t}||S_{t}\right] \end{array}$$ (11)

**Figure 4:**
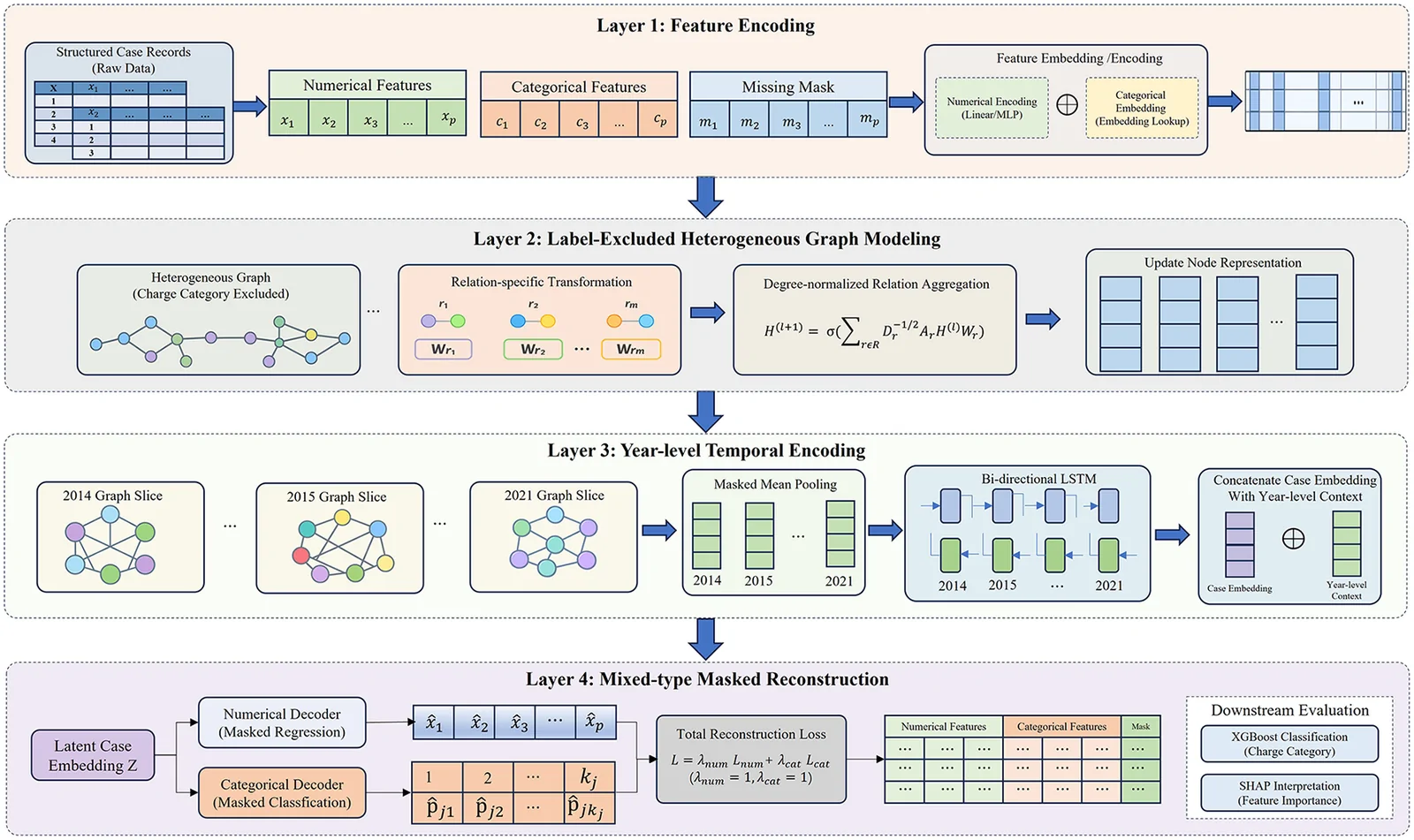
Architecture of the proposed HetGConv-TAE model with label-excluded graph construction, relation-normalized aggregation, year-level bidirectional LSTM temporal encoding, mixed-type masked reconstruction, and downstream charge classification after imputation.

The decoder then reconstructed the structured attributes of each case:


$$\begin{array}{l} {\hat{X}}_{i,t}=g_{\phi }\left({\tilde{\text{h}}}_{i,t}\right) \end{array}$$ (12)

where *g*_ϕ_ denotes the decoder. This design allows temporal variation to be modeled at the yearly context level while retaining case specific heterogeneous graph information for missing value reconstruction. For categorical variables, cross entropy loss was used. A general mixed reconstruction loss can be expressed as


$$\begin{array}{l} L=\lambda_{num}\underset{\left(i,j\right)\in \Omega_{num}}{\sum }|x_{ij}-{\hat{x}}_{ij}|+\lambda_{cat}\underset{\left(i,j\right)\in \Omega_{cat}}{\sum }CE\left(x_{ij},{\hat{x}}_{ij}\right), \end{array}$$ (13)

where Ω_*num*_ and Ω_*cat*_ denote the sets of masked numerical and categorical entries, respectively, *CE*(·) denotes the cross-entropy loss, and λ_*num*_ and λ_*cat*_ are balancing coefficients. Figure 4 illustrates the architecture of the proposed HetGConv-TAE model. The framework begins with heterogeneous graph construction, followed by relation-aware graph aggregation, temporal encoding and decoding, and masked value reconstruction. The completed dataset is then passed to the downstream classification stage.

The mixed reconstruction loss used λ_*num*_ = 1 and λ_*cat*_ = 1. These coefficients were fixed *a priori* rather than tuned as free hyperparameters. Numerical variables were standardized before training, and categorical cross entropy losses were averaged across variable groups. Equal weighting was therefore adopted to prevent either numerical or categorical reconstruction from being manually emphasized in the optimization objective.

### 2.4 Experimental design and downstream charge classification

The experimental design follows the proposed analytical pipeline. After judicial documents are converted into structured person-level observations, missing entries are reconstructed using different imputation methods. The completed datasets are then passed to the same downstream XGBoost classifier. This design evaluates the framework from two linked perspectives: upstream reconstruction quality and downstream charge classification performance after reconstruction.

Controlled missingness experiments were conducted under 10%, 20%, and 30% missing rates. These settings represent mild, moderate, and relatively severe incompleteness. The same missingness settings were applied to all methods to ensure fair comparison. These controlled settings were used as standardized benchmark experiments for comparing imputation methods. They were not intended to reproduce the full natural missingness mechanism in the original judicial records, which was examined separately through the missingness mechanism analysis described in Section 2.2. The reconstruction performance of HetGConv-TAE was evaluated against both conventional and graph-based imputation methods. The benchmark set included MICE, KNN, GAIN, and GAE, together with three stronger temporal and relational graph baselines, namely GRIN, SPIN, and a multi-relational temporal graph neural network baseline, denoted as MR-TGNN. These additional baselines were introduced to examine whether the performance advantage of HetGConv-TAE remained when temporal dependency and relational graph structure were explicitly considered. All methods were evaluated using the same controlled missingness settings, predictor sets, random seeds, missingness masks, and data partitions to ensure a fair comparison. The main model hyperparameters, training settings, software environment, hardware configuration, and code availability information are reported in Table 5.

**Table 5:** Reproducibility settings of the experimental framework.

Category	Settings and configuration
Data strategy	Stratified split, 80% training and 20% testing; 5-fold cross-validation
Random seeds	2021, 2022, 2023, 2024, and 2025
HetGConv-TAE	Two heterogeneous layers; hidden dimension: 64; latent dimension: 32; ReLU activation; dropout: 0.2; static degree-normalized relation weights; no charge-category nodes or charge-derived edges
Training hyperparameters	Optimizer: Adam; learning rate: 0.001; weight decay: 1e-4; epochs: 200; patience: 20
Baseline setup	MICE, KNN, GAIN, GAE, GRIN, SPIN, and MR-TGNN evaluated under identical masks, seeds, data partitions, and label-excluded predictor sets.
Downstream XGBoost	n_estimators: 500; max_depth: 6; learning_rate: 0.05; subsample: 0.8
Environment	Python 3.9; PyTorch 2.0; PyG 2.3; Scikit-learn 1.2; XGBoost 1.7
Hardware	Intel Xeon Gold 6248R; NVIDIA RTX 3090 with 24 GB memory; 128 GB RAM; Ubuntu 20.04
Code availability	Code will be released upon publication through an anonymized repository
Graph construction	15,184 case nodes; 124 non-target attribute nodes; 57,699 non-target case-attribute edges after excluding charge-category nodes and case-charge edges
Temporal alignment	Yearly slices from 2014 to 2021; masked pooling to fixed-dimensional slice vectors; bidirectional LSTM year-context branch
Loss weights	lambda_num = 1.0 and lambda_cat = 1.0 after numerical standardization and categorical-loss averaging

This experimental design distinguishes empirical missingness diagnosis from benchmark evaluation. The original missingness analysis was used to examine whether the judicial records were consistent with MCAR, MAR, or context-dependent missingness, whereas the controlled masks were used as standardized benchmark masks for comparing methods under matched missingness severity. Thus, the synthetic masks should not be interpreted as a full simulation of the natural missingness mechanism.

Table 5 summarizes the reproducibility settings of the experimental framework. HetGConv-TAE and all baseline methods were evaluated under the same missingness masks, random seeds, and data partition settings. The downstream XGBoost classifier was trained on each reconstructed dataset using the same evaluation protocol. This design ensured that performance differences were attributable to reconstruction quality rather than differences in random initialization, data split, or missingness mask generation.

Upstream reconstruction performance was evaluated using Mean Absolute Error (MAE) for numerical entries and Correct Imputation Rate (CIR) for categorical entries. Downstream classification performance was evaluated using weighted Precision, weighted Recall, weighted F1 score, G-Mean, and ROC-AUC. Macro F1 was reported separately in the ablation analysis. Mean Absolute Error measures numerical reconstruction deviation, while Correct Imputation Rate evaluates the proportion of correctly recovered categorical entries. Ablation experiments were conducted to examine the contribution of different components of HetGConv-TAE. In addition to two main reduced variants that removed the heterogeneous graph convolution module or the temporal modeling component, this study further designed finer grained ablations on relation types, node groups, and aggregation components. The relation type ablations removed administrative district relations, place category relations, drug-related relations, and co-offending relations. The node group ablation removed offender profile nodes, including demographic and prior record attributes. The aggregation ablation replaced relation-aware aggregation with shared homogeneous aggregation. These experiments were used to evaluate whether graph structure, temporal information, substance-evidence relations, urban contextual relations, offender-profile attributes, case-organization relations, temporal relations, and relation-specific message passing contributed to missing data reconstruction and downstream charge classification.

After missing data reconstruction, the completed datasets were used for downstream drug crime charge classification. XGBoost was selected as the classifier because it performs well on structured tabular data, captures non-linear feature interactions, and is relatively robust under mixed feature types and imbalanced class distributions (Chen and Guestrin, 2016). In this study, XGBoost was not used as an independent methodological contribution. Instead, it served as a downstream evaluator to test whether improved reconstruction quality could be translated into better charge classification performance. Let ${ŷ}_{i}^{\left(t\right)}$ denote the predicted output for sample *i* at boosting iteration *t*. The general objective function of XGBoost can be written as


$$\begin{array}{l} J^{\left(t\right)}=\overset{n}{\underset{i=1}{\sum }}l\left(y_{i},{ŷ}_{i}^{\left(t-1\right)}+f_{t}\left({\text{x}}_{i}\right)\right)+\Omega \left(f_{t}\right), \end{array}$$ (14)

where *l*(·) denotes the training loss, *f*_*t*_(·) denotes the regression tree added at iteration *t*, and Ω(*f*_*t*_) denotes the regularization term. A common regularization form is


$$\begin{array}{l} \Omega \left(f_{t}\right)=\gamma T+\frac{1}{2}\lambda \overset{T}{\underset{k=1}{\sum }}w_{k}^{2}, \end{array}$$ (15)

where *T* is the number of leaf nodes, *w*_*k*_ denotes the score of leaf *k*, and γ and λ are regularization parameters. The same stratified training and testing split was used across all reconstructed datasets. This design ensured that performance differences were attributable to reconstruction quality rather than random partition variation. In addition, all experiments were repeated under the five random seeds reported in Table 5. The same seeds, missingness masks, and data splits were applied to all imputation methods and downstream classifiers to support reproducible and comparable evaluation. Model performance was evaluated using Precision, Recall, F1 score, G-Mean, and ROC-AUC. These metrics were selected because the charge categories were highly imbalanced, and accuracy alone would be insufficient to evaluate classification quality. To reduce information leakage, the reconstruction and classification workflow was designed under training and testing separation. The downstream classifier was trained only on the training portion of each reconstructed dataset and evaluated on the corresponding test portion. Target labels and variables that directly revealed the charge category were excluded from predictors. Judicial identifiers and charge related textual fields were therefore not used as input features in the classifier.

The same split-aware and label-excluded principle was applied to the imputation stage. HetGConv-TAE was trained using non-target observed attributes and observation masks, and target labels or charge-derived graph edges were not available to the graph convolution, temporal encoder, decoder, or reconstruction loss. This setting aligns the unsupervised reconstruction task with the downstream evaluation protocol.

### 2.5 Model interpretation

SHAP was used to interpret the final XGBoost classifier trained on the HetGConv-TAE completed dataset. SHAP is based on Shapley value theory and assigns each feature a contribution value for a given prediction by considering its marginal contribution across possible feature coalitions (Lundberg and Lee, 2017; Jin et al., 2024). This approach is suitable for the present study because it provides both global and local explanations for model behavior. For a prediction function *f*(·), the SHAP value of feature *j* can be written as


$$\begin{array}{l} \phi_{j}=\underset{S\subseteq F\backslash \left\{j\right\}}{\sum }\frac{|S|!\left(|F|-|S|-1\right)!}{|F|!}\left[f\left(S\cup \left\{j\right\}\right)-f\left(S\right)\right], \end{array}$$ (16)

where *F* denotes the full feature set, *S* denotes a subset of features excluding feature *j*, and ϕ_*j*_ denotes the contribution of feature *j* to the prediction. In this study, SHAP was used to examine global feature importance, model feature dependence, and class specific contribution patterns of key variables such as drug weight, place category, administrative district, co-offending status, age, prior record, and drug type. SHAP explanations were interpreted as predictive contribution evidence rather than causal effects. This distinction is important because judicial charge classification may be shaped by legal rules, case facts, local enforcement practices, and unobserved contextual factors. Therefore, SHAP results were used to evaluate whether the model relied on legally meaningful and contextually plausible predictors, not to infer direct legal or behavioral causality.

## 3 Results

### 3.1 Descriptive structure, missingness patterns, and feature dependency

Before evaluating the proposed reconstruction model, the structural characteristics of the judicial dataset were examined to clarify the empirical basis for subsequent imputation and classification. As reported in Section 2, the final analytical dataset contained 15,184 person-level observations transformed from 12,620 valid judicial documents. The descriptive results showed clear temporal fluctuation, spatial heterogeneity, drug type imbalance, and charge category imbalance. Methamphetamine related cases formed the dominant drug type, while smuggling, trafficking, transporting, or manufacturing drugs accounted for the largest share of charge categories. These patterns indicate that the dataset reflects not only legal outcome imbalance but also uneven distributions across time, urban districts, and offense contexts.

In addition to descriptive imbalance, the dataset exhibited non-trivial missingness across multiple structured variables. Figure 3 shows the variable-wise missingness distribution in the structured drug crime dataset. Missing values were unevenly distributed across features and affected several legally and contextually relevant variables. This pattern indicates that missingness was not limited to peripheral attributes. Instead, incomplete entries appeared in variables that may directly influence charge classification, such as offender characteristics, spatial context, and case related features.

To further assess whether the original missing values were completely random, missingness mechanism analyses were conducted for variables with incomplete entries. As reported in Table 6, substance evidence missingness was associated with charge category (χ^2^ = 38.52, *p* < 0.01) and drug type (*p* < 0.05), showing that substance-evidence missingness varied across observed charge categories and drug types. Event space context missingness was associated with year (χ^2^ = 54.16, *p* < 0.001) and district (*p* < 0.01), showing that event-space-context missingness varied across observed years and administrative districts. Offender profile missingness was associated with co-offending status (χ^2^ = 18.24, *p* < 0.01) and age group (*p* < 0.05), showing that offender-profile missingness varied across observed co-offending-status and age groups. In addition, the overall Little's MCAR test was significant (χ^2^ = 142.64, *p* < 0.001), rejecting the assumption that the original missingness pattern was completely at random.

**Table 6:** Original missingness mechanism analysis.

Missing variable group	Key associations & statistical test	Inferred mechanism & empirical context
Substance evidence	Charge category (χ^2^ = 38.52, *p* < 0.01); Drug type (*p* < 0.05)	Evidence consistent with context-dependent or MAR-like missingness: missingness varies across observed charge categories and drug types.
Event space context	Year (χ^2^ = 54.16, *p* < 0.001); District (*p* < 0.01)	Evidence consistent with context-dependent or MAR-like missingness: missingness is associated with observed year and administrative district.
Offender profile	Co-offending (χ^2^ = 18.24, *p* < 0.01); Age group (*p* < 0.05)	Evidence consistent with context-dependent or MAR-like missingness: missingness is associated with observed co-offending status and age group.
Overall dataset	Overall Little's MCAR test:χ^2^ = 142.64, *p* < 0.001	MNAR cannot be ruled out: latent, unobserved recording practices may also contribute to the overall missingness pattern.

Little's MCAR test (Little, 1988) rejects the hypothesis that the overall missingness pattern is completely random. The reported associations indicate systematic dependence on observed variables but do not by themselves prove MAR or exclude MNAR. Controlled MCAR masks are used only for standardized benchmark comparison.

These results reject a pure MCAR interpretation of the original judicial records and indicate that missingness is systematically associated with several observed legal, temporal, spatial, and offender-related variables. Such associations are consistent with context-dependent or MAR like missingness. However, they do not provide definitive identification of the missingness mechanism, because MNAR cannot be ruled out when missingness may also depend on unobserved clerical practices, documentary completeness, evidentiary availability, or institutional recording processes. Accordingly, the empirical missingness analysis and the controlled benchmark experiments should be interpreted as complementary rather than interchangeable analyses. The former characterizes departures from pure MCAR in the original records, whereas the latter provides a standardized and reproducible basis for comparing imputation methods under matched missingness severity. Performance under the controlled masks should therefore not be interpreted as direct validation of the model under MAR or MNAR conditions. Therefore, the present study treats incomplete judicial records as a structured data quality problem and uses controlled missingness experiments only as standardized benchmarks for model comparison.

**Figure 5:**
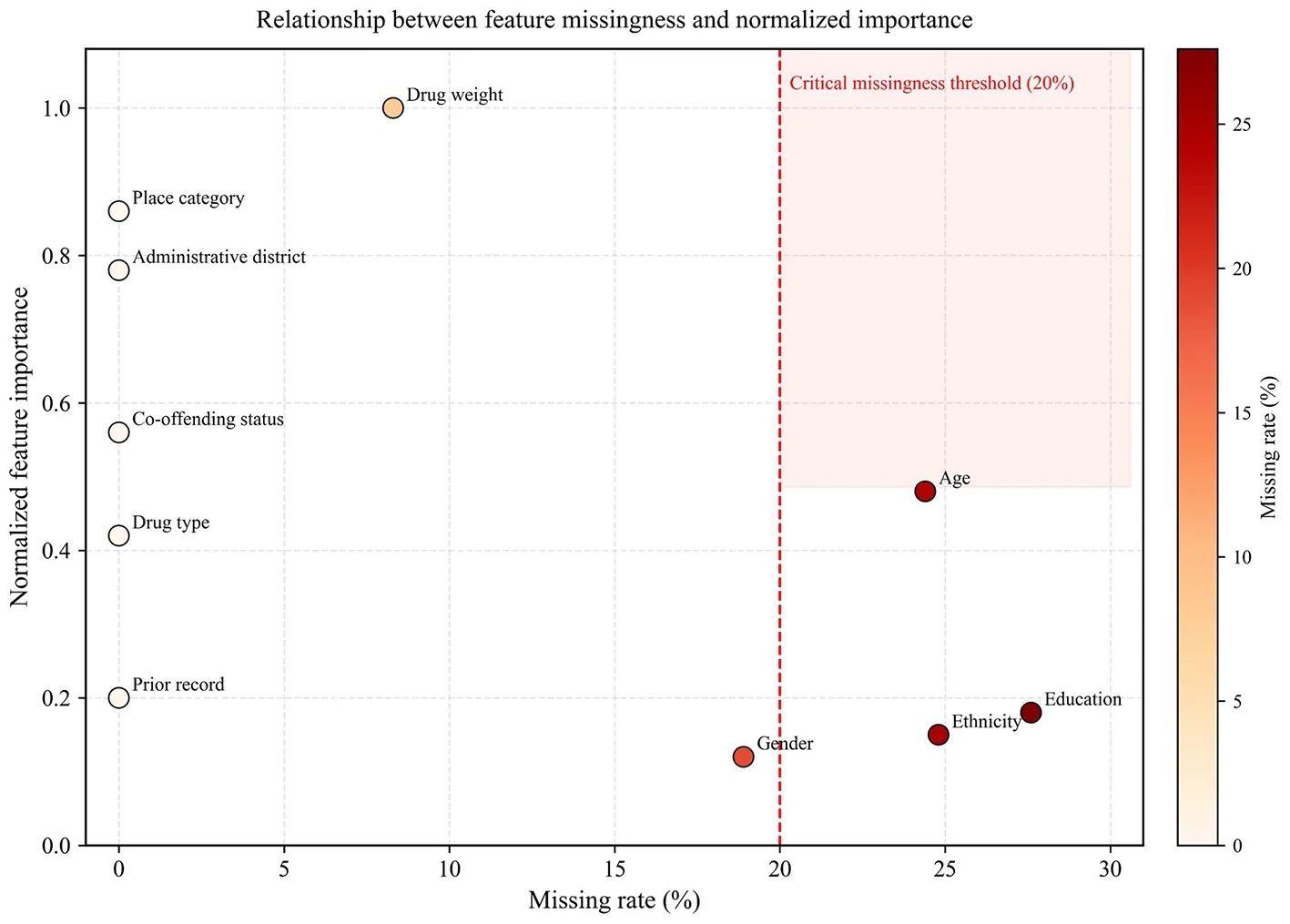
Relationship between feature missingness and normalized importance in the final classification framework.

To further examine whether missing values occurred in analytically important variables, missingness rates were compared with normalized feature importance in the final classification framework. Figure 5 presents the relationship between feature missingness and normalized importance. The results show that several variables with non-negligible predictive importance also exhibited missingness. In particular, age had both a relatively high missing rate and measurable predictive contribution, while drug weight remained one of the most influential variables despite a lower missing rate. This finding suggests that incomplete data may distort key predictive signals rather than only affecting secondary descriptors.

**Figure 6:**
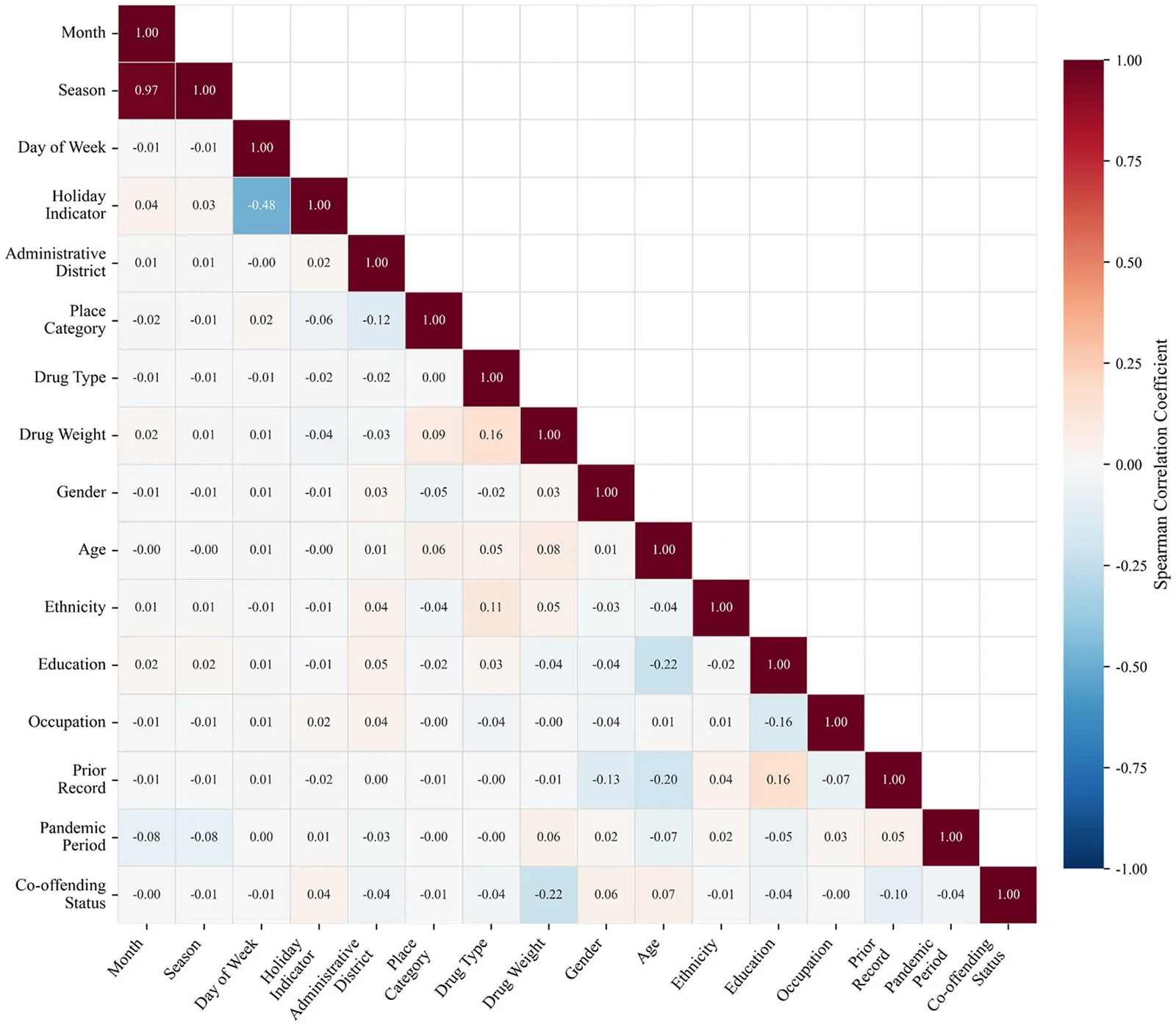
Spearman correlation matrix of structured features in the judicial dataset.

The dependency structure among structured variables was also examined. Figure 6 presents the Spearman correlation matrix of the main structured features in the judicial dataset. Although most pairwise correlations were weak or moderate, nonzero pairwise associations were observed among several temporal and contextual variables. These correlations provide motivation for examining relational structure, but they do not by themselves establish statistical dependence or causality. This pattern supports the use of a graph-based representation to capture shared relations among case records and attribute entities.

Taken together, the evidence from Figures 3, 5, and 6 indicates that the dataset was incomplete, imbalanced, and relationally structured. These characteristics form the empirical basis for the proposed HetGConv-TAE model and explain why a reconstruction-aware framework is required before downstream charge classification.

### 3.2 Imputation performance and ablation analysis

Table 7 reports the reconstruction performance of different imputation methods under 10%, 20%, and 30% missingness. The results are reported as mean ± standard deviation across repeated random seeds. HetGConv-TAE consistently achieved the lowest MAE and the highest CIR across all missingness settings. Under 10%, 20%, and 30% missingness, HetGConv-TAE achieved MAE values of 7.223 ± 0.06, 7.312 ± 0.07, and 7.278 ± 0.07, respectively. It also achieved CIR values of 0.713 ± 0.01, 0.724 ± 0.01, and 0.716 ± 0.01, respectively. Paired significance tests under this repeated-seed protocol indicated consistent differences, but the absolute margins were moderate and should be interpreted within the evaluated benchmark setting rather than as evidence of general dominance over all temporal graph imputation models.

**Table 7:** Imputation performance of different methods under repeated random seeds.

Imputation method	10% MAE	10% CIR	20% MAE	20% CIR	30% MAE	30% CIR
MICE	8.314 ± 0.12	0.694 ± 0.02	8.932 ± 0.15	0.666 ± 0.02	9.016 ± 0.18	0.651 ± 0.02
KNN	7.964 ± 0.09	0.576 ± 0.03	7.903 ± 0.11	0.541 ± 0.03	8.031 ± 0.13	0.530 ± 0.03
GAIN	8.672 ± 0.14	0.643 ± 0.02	8.919 ± 0.16	0.630 ± 0.02	9.519 ± 0.20	0.623 ± 0.02
GAE	7.485 ± 0.08	0.621 ± 0.02	7.493 ± 0.09	0.634 ± 0.02	7.704 ± 0.10	0.624 ± 0.02
HetGConv-TAE	7.223 ± 0.06^*^	0.713 ± 0.01^*^	7.312 ± 0.07^*^	0.724 ± 0.01^*^	7.278 ± 0.07^*^	0.716 ± 0.01^*^

Values are reported as mean ± standard deviation across repeated random seeds. ^*^ indicates paired statistical significance against the strongest evaluated baseline under the same missingness setting; effect sizes should be interpreted within the repeated-seed benchmark protocol.

Among the original benchmark methods, GAE was the strongest alternative in terms of MAE, but it remained inferior to HetGConv-TAE across all missingness levels. This result suggests that graph representation alone is useful but insufficient for fully reconstructing incomplete judicial records. MICE and GAIN showed more noticeable deterioration as missingness increased, indicating weaker robustness when structured judicial attributes became sparse. KNN also performed less competitively, likely because local similarity in a flat feature space was insufficient to capture the semantic and contextual relations embedded in judicial case data.

The observed performance advantage of HetGConv-TAE is consistent with the structure of the dataset. Judicial records are connected through shared drug types, place categories, administrative districts, offender characteristics, and temporal contexts. Conventional imputation methods generally rely on conditional dependence, local similarity, or distributional reconstruction, but they do not explicitly represent these heterogeneous relations. HetGConv-TAE improves reconstruction by integrating relation-aware graph aggregation with temporal encoding. This joint inductive bias is well-aligned with the relational and temporal structure of incomplete judicial records.

**Figure 7:**
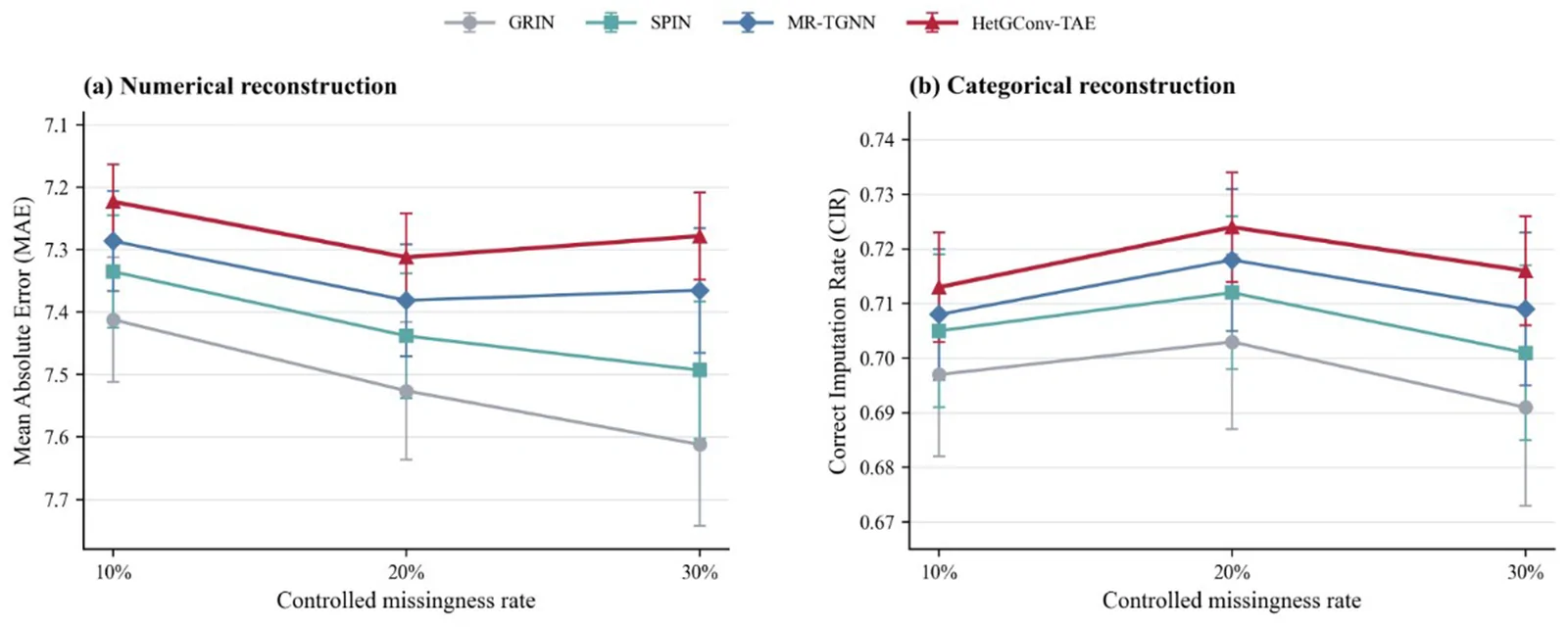
Reconstruction performance comparison with stronger temporal and relational graph imputation methods under different controlled missingness rates. **(a)** Numerical reconstruction performance evaluated by Mean Absolute Error. **(b)** Categorical reconstruction performance evaluated by Correct Imputation Rate. Error bars represent ±1 standard deviation across five matched random seeds.

To further evaluate whether the reconstruction advantage remained when compared with stronger temporal and relational graph imputation methods, GRIN, SPIN, and MR-TGNN were additionally evaluated under the same experimental protocol. Figure 7 compares their reconstruction performance with HetGConv-TAE under 10%, 20%, and 30% controlled missingness.

As shown in Figure 7a, HetGConv-TAE achieved the lowest mean MAE across all three missingness levels, while MR-TGNN was consistently the closest competing method, followed by SPIN and GRIN. A similar pattern was observed in Figure 7b, where HetGConv-TAE maintained the highest mean CIR across the three missingness settings. Although the performance margins over the stronger graph-based baselines were moderate, HetGConv-TAE consistently ranked first across both reconstruction metrics and all missingness levels. These results suggest that the joint modeling of heterogeneous relations and temporal context provides consistent additional reconstruction benefits within the evaluated benchmark setting beyond generic temporal or multi-relational graph propagation.

The fine-grained ablation results are reported in Table 8. The full HetGConv-TAE model achieved the best upstream reconstruction performance, with an MAE of 7.223 and a CIR of 0.713, and the best downstream classification performance, with a Macro F1 of 78.50% and a ROC-AUC of 86.20%. Removing the entire heterogeneous graph convolution module produced the largest performance decline, with the MAE increasing to 8.618 and the CIR decreasing to 0.602. The downstream Macro F1 and ROC-AUC also decreased to 71.40% and 79.50%, respectively. Removing the year-level temporal autoencoding branch also reduced performance, with an MAE of 7.954, a CIR of 0.645, a Macro F1 of 74.80%, and a ROC-AUC of 82.10%. These results indicate that both heterogeneous graph structure and temporal variation are important for reconstructing incomplete judicial records.

**Table 8:** Fine-grained ablation results of HetGConv-TAE under 10% controlled missingness.

Model variant and modification	Upstream reconstruction MAE/CIR	Downstream classification Macro F1/ROC-AUC
Full HetGConv-TAE: no component removed	7.223/0.713	78.50%/86.20%
Without graph convolution: remove the heterogeneous graph module	8.618/0.602	71.40%/79.50%
Without temporal autoencoding: remove the year-level temporal encoder branch	7.954/0.645	74.80%/82.10%
Without district relation: remove administrative district spatial edges	7.485/0.692	76.90%/84.60%
Without place relation: remove place category urban contextual edges	7.421/0.696	77.20%/85.00%
Without drug relation: remove drug type and drug weight substance-evidence edges	7.652/0.671	75.50%/83.40%
Without offender profile nodes: remove demographic and prior record attribute nodes	7.314/0.705	78.00%/85.70%
Without co-offending relation: remove co-offending social network edges	7.289/0.709	78.20%/85.90%
Without relation-aware aggregation: replace with shared homogeneous aggregation	7.543/0.684	76.10%/84.00%

The relation and node group ablations show that different semantic components contributed unequally to the model. Removing drug-related relations reduced the Macro F1 to 75.50% and the ROC-AUC to 83.40%, indicating that drug type and drug weight relations are closely related to downstream charge classification. Removing administrative district relations or place category relations also weakened performance, suggesting that spatial and urban contextual information supports missing value recovery. Removing offender profile nodes and co-offending relations caused smaller decreases, indicating that these components provide additional but less dominant information. Replacing relation-aware aggregation with shared homogeneous aggregation increased the MAE to 7.543. The CIR to 0.684, the Macro F1 to 76.10%, and the ROC-AUC to 84.00%. This result shows that explicitly modeling relation types is more effective than treating all graph connections as homogeneous.

The reconstruction results demonstrate that the observed advantage of HetGConv-TAE was not limited to a single missingness level. Its performance remained stable under mild, moderate, and relatively severe incompleteness. This stability is important for judicial and public safety data analytics because missing values in real world legal archives often arise from heterogeneous documentary narratives, inconsistent reporting standards, and structured coding errors. Therefore, the proposed reconstruction framework provides a more reliable input representation for downstream charge classification.

### 3.3 Downstream charge classification performance

Table 9 reports the downstream classification performance of XGBoost trained on datasets reconstructed by different imputation methods under 10%, 20%, and 30% missingness. The results are reported as mean ± standard deviation across repeated random seeds, with each imputation method reported in a separate row. Compared with datasets reconstructed by MICE, KNN, GAIN, and GAE, the dataset reconstructed by HetGConv-TAE produced the best downstream classification performance within the evaluated baseline set. HetGConv-TAE achieved F1 scores of 80.07% ± 0.38, 80.83% ± 0.42, and 80.14% ± 0.45 under 10%, 20%, and 30% missingness, respectively. It also achieved ROC-AUC values of 90.54% ± 0.25, 91.50% ± 0.31, and 91.48% ± 0.33, respectively. These results suggest that improved reconstruction quality was reflected in downstream charge classification under the fixed XGBoost evaluation protocol, while the practical magnitude of the gain should be interpreted together with the reported standard deviations.

**Table 9:** Classification performance of XGBoost based on different reconstructed datasets under repeated random seeds.

Missingness (%)	Imputation method	Weighted F1 score	ROC-AUC
10	MICE	74.4% ± 0.5	80.1% ± 0.4
10	KNN	78.1% ± 0.5	89.0% ± 0.4
10	GAIN	78.4% ± 0.5	88.9% ± 0.4
10	GAE	79.0% ± 0.5	89.4% ± 0.4
10	HetGConv-TAE	80.07% ± 0.38^*^	90.54% ± 0.25^*^
20	MICE	74.3% ± 0.6	80.0% ± 0.4
20	KNN	77.7% ± 0.6	88.9% ± 0.4
20	GAIN	78.1% ± 0.6	88.9% ± 0.4
20	GAE	78.7% ± 0.6	89.5% ± 0.4
20	HetGConv-TAE	80.83% ± 0.42^*^	91.50% ± 0.31^*^
30	MICE	74.5% ± 0.6	80.0% ± 0.5
30	KNN	77.6% ± 0.6	89.1% ± 0.5
30	GAIN	78.1% ± 0.6	88.7% ± 0.5
30	GAE	79.3% ± 0.6	89.5% ± 0.5
30	HetGConv-TAE	80.14% ± 0.45^*^	91.48% ± 0.33^*^

Values are reported as mean ± standard deviation across repeated random seeds. ^*^ indicates paired statistical significance against the strongest evaluated baseline under the same missingness setting; effect sizes should be interpreted within the fixed-XGBoost benchmark protocol.

At 10% missingness, XGBoost trained on the HetGConv-TAE reconstructed dataset achieved 80.07% Precision, 80.22% Recall, 80.07% F1 score, 69.58% G-Mean, and 90.54% ROC-AUC. At 20% missingness, the same pipeline achieved 80.78% Precision, 80.06% Recall, 80.83% F1 score, 69.83% G-Mean, and 91.50% ROC-AUC. At 30% missingness, the model remained stable, with 80.14% Precision, 80.32% Recall, 80.14% F1 score, 69.64% G-Mean, and 91.48% ROC-AUC.

These results indicate that HetGConv-TAE based reconstruction provided a moderately more discriminative feature representation for downstream charge classification than the evaluated baseline methods. The performance gains were observed not only in conventional classification metrics such as Precision, Recall, and F1 score, but also in G-Mean and ROC-AUC, which are more informative under imbalanced charge distributions. This is important because the dataset contains a dominant majority class and several smaller charge categories. In such a setting, high overall accuracy alone would not be sufficient to demonstrate reliable classification performance.

The downstream results are consistent with the reconstruction results reported in Table 7. Better reconstruction quality improved not only numerical fidelity but also the effective separability of charge categories. This suggests that the completed feature matrix retained information that was meaningful for judicial classification rather than merely reducing reconstruction error. The result is especially relevant for imbalanced legal prediction tasks, where minority class signals may already be weak and can be further diluted by missingness.

Because the same stratified train test split and the same XGBoost settings were used across reconstructed datasets, the observed differences in downstream performance can be more plausibly attributed to reconstruction quality rather than classifier retuning or favorable data partitioning. The observed downstream advantage of the HetGConv-TAE and XGBoost pipeline therefore indicates that upstream reconstruction quality influenced downstream legal data analytics under the fixed-classifier protocol; it does not by itself establish classifier-independent superiority.

### 3.4 SHAP-based interpretation of charge classification

**Figure 8:**
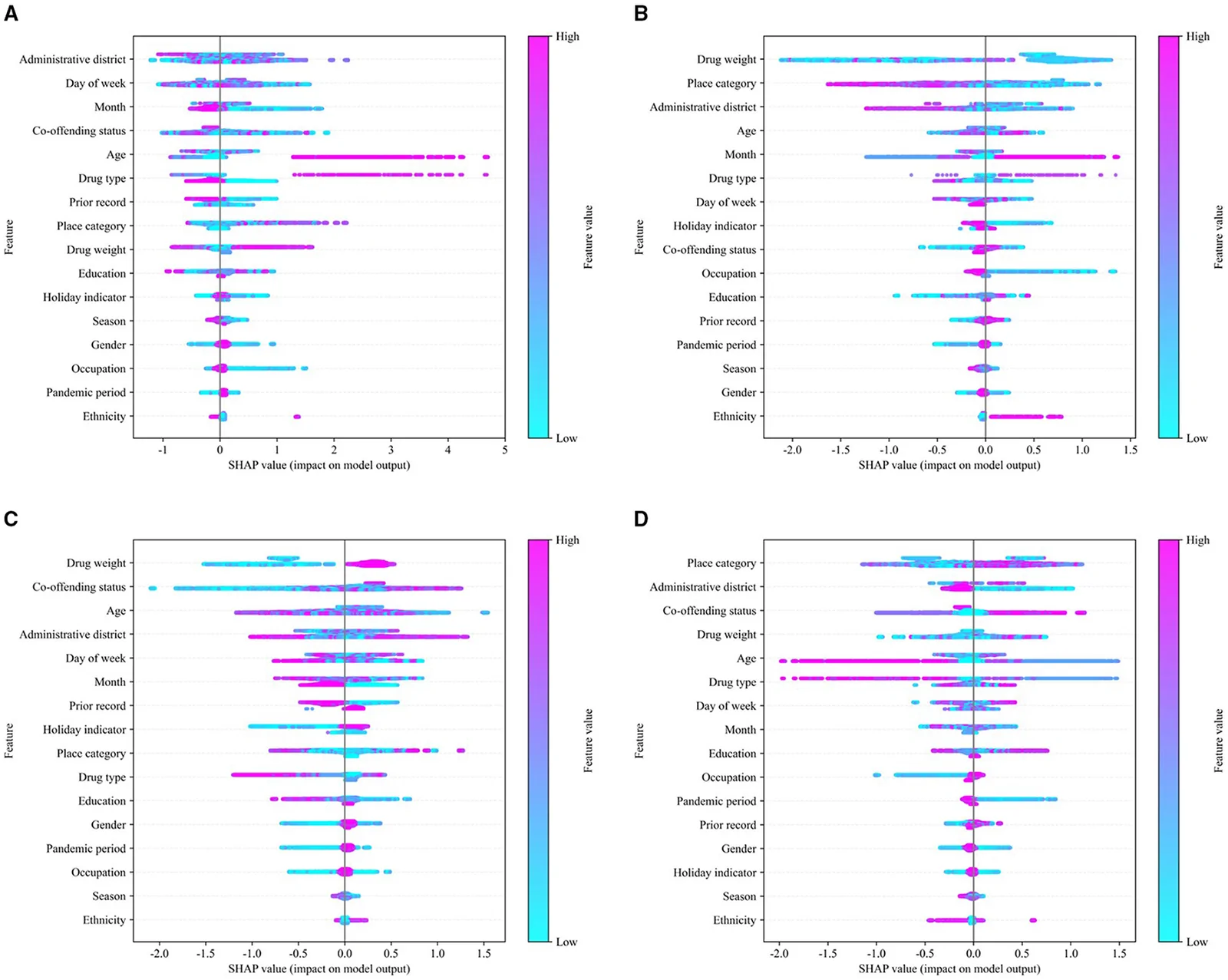
Class specific SHAP summary plots for the four charge categories based on the XGBoost classifier trained on HetGConv-TAE reconstructed data. **(A)** SHAP summary plot for smuggling, trafficking, transporting, or manufacturing drugs. **(B)** SHAP summary plot for sheltering others to take drugs. **(C)** SHAP summary plot for illegal possession of drugs. **(D)** SHAP summary plot for other charges.

**Figure 9:**
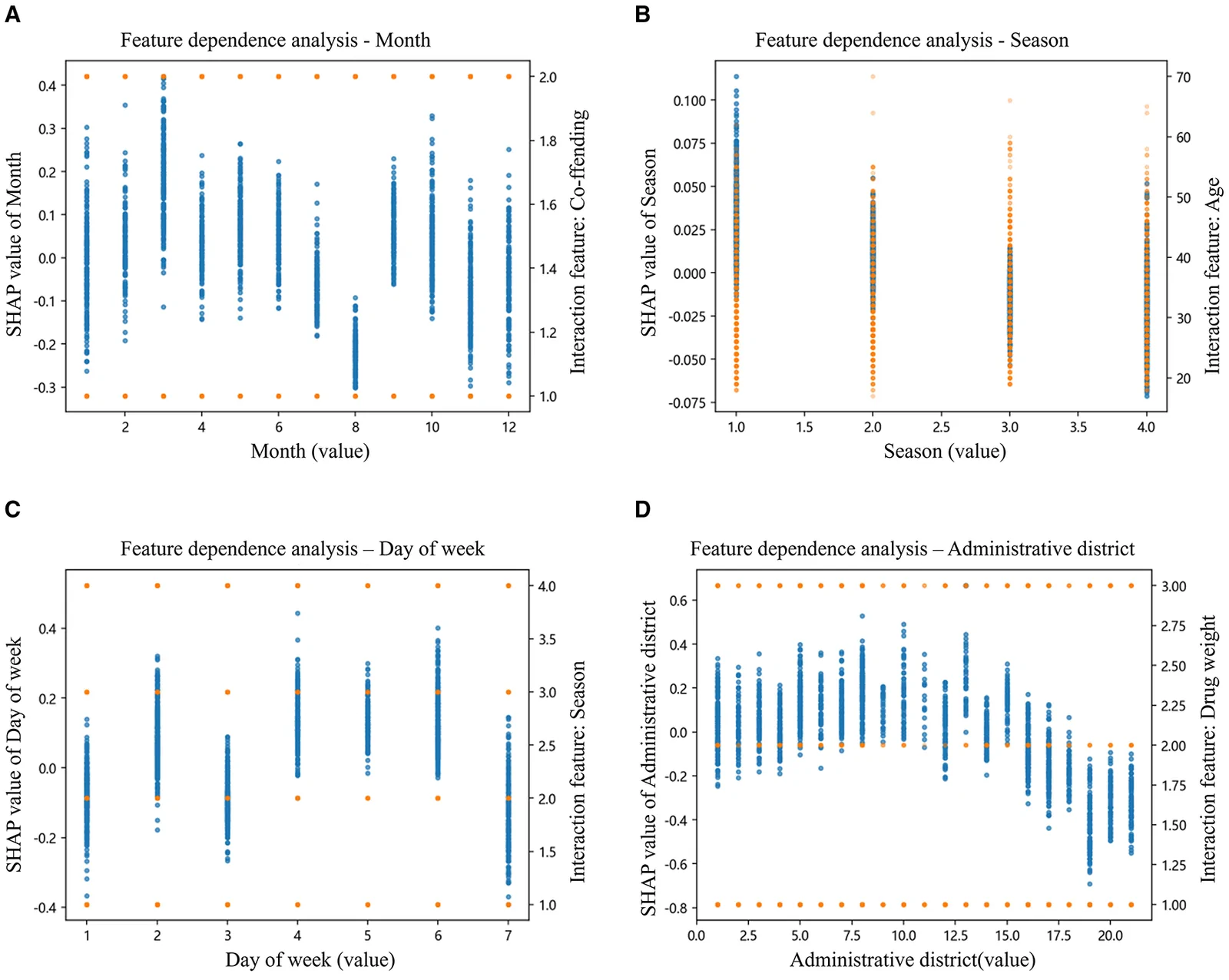
SHAP dependence plots for selected contextual variables in the XGBoost classifier trained on the HetGConv-TAE completed dataset. **(A)** month; **(B)** season; **(C)** day of week; **(D)** administrative district.

To examine whether the final classifier relied on substantively meaningful predictors, SHAP was used to interpret the XGBoost model trained on the HetGConv-TAE completed dataset. Figure 8 presents the class-specific SHAP summary plots for the four charge categories, and Figures 9A–D further show feature dependence plots for four representative contextual variables, namely month, season, day of week, and administrative district. Together, these results provide both a global view of feature importance and a more detailed description of how selected variables influence model output across different value ranges.

As shown in Figure 8, drug weight, place category, and administrative district were consistently among the most influential predictors across charge categories. These variables are legally relevant and contextually interpretable, suggesting that the classifier did not rely only on incidental statistical patterns. The contribution structure also differed across categories. For smuggling, trafficking, transporting, or manufacturing drugs, place category, administrative district, co-offending status, and drug type showed relatively large contribution magnitudes, indicating that supply-related offenses were more closely associated with contextual and organizational characteristics. For sheltering others to take drugs, drug weight, place category, and administrative district played prominent roles, highlighting the importance of situational and spatial context. For illegal possession of drugs, drug weight was the dominant predictor, followed by co-offending status, administrative district, and age, which is consistent with the legal relevance of drug quantity and individual case circumstances. For the residual category of other charges, the SHAP pattern was more dispersed, with administrative district, day of week, age, and month showing relatively balanced effects, reflecting the heterogeneous nature of this category.

Figures 9A–D further illustrate how temporal and spatial variables contributed to charge classification. Figure 9A shows that the SHAP contribution pattern associated with Month was non-monotonic, with positive contributions concentrated in several months, particularly around March and October, whereas August and several late year months exhibited denser negative contributions. Figure 9B shows that season related SHAP contribution patterns were also heterogeneous, with Season 1 associated with more positive contributions and Seasons 3 and 4 showing denser negative contributions. Figure 9C shows differentiated contribution patterns across days of the week, while Figure 9D indicates substantial spatial heterogeneity across administrative districts.

Taken together, the SHAP results indicate that the reconstructed feature space preserved meaningful discriminative information embedded in both contextual and offense-related variables. The repeated importance of place category and administrative district suggests that urban context contributed substantially to charge classification, while the strong contribution of drug weight is consistent with its legal relevance for distinguishing offense seriousness and charge type. These findings support both the predictive effectiveness and the substantive plausibility of the proposed framework.

It should be noted that SHAP values represent predictive contributions within the trained classifier rather than causal effects. Accordingly, the observed feature importance patterns should be interpreted as reflecting how the model distinguished among charge categories, rather than as evidence that any individual factor directly determines legal outcomes. This caution is especially important for temporal variables such as Month and Season, whose SHAP dependence patterns may reflect institutional timing and record-generation processes as well as social activity patterns.

## 4 Discussion

### 4.1 Principal findings

This study developed a reconstruction-aware urban crime analytics framework for drug crime charge classification from incomplete judicial records. The empirical analysis based on judicial documents from Chengdu showed that incomplete legal archives can be transformed into structured person-level data and further reconstructed through heterogeneous graph temporal learning. The results provide three main findings.

The first finding is that incomplete judicial records should be treated as a core data quality problem rather than as a routine preprocessing issue. The missingness analysis showed that missing entries were unevenly distributed across variables and affected several features with legal or contextual relevance. This pattern implies that missing values may weaken the representation of important case facts, offender characteristics, spatial context, and temporal conditions. If these missing values are removed or handled through simple imputation, the resulting data may distort the feature space used for downstream charge classification.

The second finding is that HetGConv-TAE improved missing data reconstruction under different levels of incompleteness. Compared with MICE, KNN, GAIN, and GAE, the proposed model achieved the best reconstruction performance within the evaluated baseline set under 10%, 20%, and 30% standardized missingness masks. The performance advantage indicates that judicial records are better represented as heterogeneous relational data rather than as independent rows in a conventional tabular matrix. Case observations are connected through shared drug types, place categories, administrative districts, offender profiles, and temporal contexts. By jointly modeling these relations and their temporal evolution, HetGConv-TAE was able to recover incomplete entries more effectively than methods relying only on conditional dependence, local similarity, adversarial reconstruction, or homogeneous graph encoding.

The third finding is that improved reconstruction quality translated into better downstream charge classification. XGBoost trained on the HetGConv-TAE completed dataset achieved the best performance among the evaluated reconstructed datasets, with F1 scores close to 80% and ROC-AUC values above 90%. This result indicates that reconstruction quality is not only an upstream technical metric but also a determinant of downstream predictive reliability. In imbalanced legal prediction tasks, where minority charge categories may contain limited discriminative signals, preserving the structure of incomplete records is especially important.

The SHAP-based interpretation further showed that the model relied primarily on legally meaningful and contextually plausible variables, including drug weight, place category, administrative district, co-offending status, drug type, and age. Drug weight represents a key indicator of offense seriousness. Place category captures the situational environment of drug crime. Administrative district reflects spatial heterogeneity in crime distribution, enforcement exposure, and urban governance context. These findings suggest that the proposed framework provides not only predictive performance but also interpretable evidence for understanding the structured relationship between legal facts and urban public safety data.

### 4.2 Methodological implications for legal and public safety data analytics

The findings have several methodological implications for legal data analytics and public safety intelligence. First, the results suggest that judicial records should not be simplified into ordinary tabular data without considering their semantic and institutional structure. Each case record is linked to multiple types of non-target entities, including offenders, substances, locations, districts, time periods, and case organization attributes; charge outcomes are reserved for downstream evaluation rather than graph construction. These entities are not independent descriptors. They jointly define the evidentiary and contextual structure of a judicial case. A heterogeneous graph representation is therefore more suitable for preserving the relational logic embedded in judicial records.

Second, the study shows that temporal variation should be incorporated into judicial data reconstruction. Drug crime case composition may change across years due to shifts in drug markets, enforcement practices, social activity patterns, court processing, and public health disruptions. The decline in case volume during 2020 and 2021 illustrates that judicial data are temporally uneven rather than stationary. A reconstruction model that ignores temporal variation may fail to capture changes in case structure and may produce less reliable imputations under non-stationary conditions. The temporal autoencoding component of HetGConv-TAE addresses this issue by using time dependent embedding sequences to support missing value recovery.

Third, the results support a task-oriented view of missing data reconstruction. Many imputation studies evaluate reconstruction only through intrinsic metrics such as MAE or classification accuracy of imputed categorical values. In contrast, the present study evaluates whether reconstructed data improve downstream charge classification. This design is important because the ultimate value of reconstruction lies not only in recovering masked entries but also in preserving information that supports meaningful analytical tasks. The consistent but moderate improvement in downstream XGBoost performance indicates that the reconstructed feature matrix retained discriminative information relevant to legal classification under the evaluated protocol.

Fourth, the framework contributes to explainable data analytics by linking reconstruction, classification, and interpretation within one workflow. In high stakes domains such as law and public safety, model performance alone is insufficient. Predictive models should also provide interpretable evidence that can be examined against domain knowledge. The SHAP results help evaluate whether the classifier relied on legally meaningful variables rather than arbitrary statistical artifacts. This interpretability layer strengthens the practical credibility of the framework and provides a basis for human review.

### 4.3 Implications for urban crime governance and social impact

The proposed framework also has practical implications for urban crime governance. Many cities have accumulated large volumes of judicial documents, police records, administrative reports, and case archives. However, these data are often difficult to use directly because they are unstructured, incomplete, uneven in quality, and inconsistent across time and administrative units. The present study shows that reconstruction-aware modeling can improve the analytical value of such archives by converting incomplete judicial records into structured and interpretable data resources.

The importance of place category and administrative district in the SHAP results indicates that judicial records can provide useful information for understanding the spatial and situational context of crime. Place category reflects the environment in which drug crime occurs, such as private residences, public venues, transportation spaces, or other urban activity settings. Administrative district captures geographic variation in case concentration, enforcement exposure, local social conditions, and governance practices. When these variables are consistently important in charge classification, they suggest that judicial records contain urban contextual signals beyond formal legal outcomes.

From a public safety perspective, the outputs of the proposed framework can be applied in four practical contexts. First, the reconstructed dataset can support judicial archive quality assessment by identifying variables and case groups with concentrated missingness. Second, it can support missing information auditing by helping analysts examine whether missing records affect the interpretation of charge patterns across districts, place categories, or time periods. Third, the completed dataset can support aggregate crime pattern analysis by comparing how different charge categories are associated with drug type, drug weight, place category, and administrative district. Fourth, the classification and SHAP results can provide auxiliary evidence for public safety research by indicating which structured variables contribute to charge category differentiation.

These applications should be limited to aggregate analysis, archival data quality improvement, and research support. The framework is not designed for automatic legal decision making, charge determination, sentencing, individual profiling, or punitive intervention. Model outputs should be interpreted as analytical support for human review rather than as substitutes for legal reasoning, evidentiary evaluation, or institutional judgment.

The framework is also relevant to data analytics for social impact because it addresses a practical problem in public sector data use. Public safety agencies often face a tension between abundant archival records and limited analytical usability. Data incompleteness, variable heterogeneity, and outcome imbalance can reduce the reliability of intelligent systems. By explicitly modeling incomplete judicial records, the proposed approach offers a pathway for improving the quality of legal data resources before they are used for prediction, explanation, or policy analysis.

The practical use of the framework should therefore follow a human reviewed and non-punitive mode. In research or administrative analysis, the model may help screen data quality problems, summarize aggregate patterns, and generate interpretable evidence for further examination. In legal or public safety institutions, any use of the model should remain secondary to human expertise, legal procedure, evidentiary standards, and institutional accountability.

### 4.4 Ethical considerations, limitations, and future research

Several ethical considerations should be emphasized. The dataset used in this study was derived from judicial documents, and the analysis was conducted at the structured record level. Even when records are publicly accessible, legal data may contain sensitive information about individuals, case facts, and social contexts. Therefore, the use of judicial records for data analytics should follow principles of data minimization, anonymization, secure storage, and responsible reporting. The analytical objective should be limited to understanding aggregate patterns and improving data quality, rather than profiling individuals or supporting automated punitive decisions.

The interpretability results should also be interpreted with caution. SHAP values quantify predictive contributions within a trained model, but they do not establish causal relationships. A high SHAP value for administrative district, place category, or offender characteristic does not mean that the feature directly causes a specific charge outcome. It only indicates that the feature contributed to the model prediction under the learned data structure. This distinction is essential because judicial outcomes are shaped by legal provisions, evidentiary standards, prosecutorial decisions, court interpretation, and unobserved contextual factors. Model explanations should therefore support critical review rather than substitute for legal reasoning. In addition, temporal SHAP patterns should be interpreted as predictive associations that may combine behavioral, administrative, enforcement, and court-processing influences, rather than as direct evidence of seasonal drug-crime behavior.

The study has several limitations. First, the empirical analysis was based on one city and one crime domain. Chengdu provides a meaningful case for urban drug crime analytics, but the generalizability of the results to other cities, other crime types, and other legal systems remains uncertain. Judicial writing styles, enforcement practices, local drug markets, and spatial governance conditions may vary across regions. Multi city validation is therefore needed to evaluate the robustness of the proposed framework.

Second, although controlled missingness experiments were conducted under 10%, 20%, and 30% missing rates, simulated missingness cannot fully reproduce all mechanisms of natural judicial data incompleteness. Real missingness may depend on case complexity, document style, evidence availability, district level reporting practices, and time specific institutional factors. Future research should incorporate more explicit missingness mechanism modeling, including comparisons between random missingness, variable dependent missingness, and context-dependent missingness.

Third, although the benchmark includes conventional imputation methods, a static graph autoencoder, and stronger temporal and relational graph baselines such as GRIN, SPIN, and MR-TGNN, the evaluation does not exhaust all contemporary graph-based imputation architectures. In addition, the downstream classifier was fixed as XGBoost to isolate the effect of reconstruction quality. This controlled design does not establish whether the relative advantage of the reconstructed representations remains unchanged across different classifier families (Cini et al., 2022; Marisca et al., 2022). Future research should therefore evaluate additional graph imputation architectures and multiple downstream classifiers.

Fourth, the current framework relies mainly on structured variables derived from judicial records. It does not yet incorporate richer external urban covariates, such as land use, points of interest, transportation networks, mobility patterns, neighborhood socioeconomic indicators, or police service activity. Integrating judicial records with external urban data may help connect charge classification more directly to urban spatial structure and public safety planning. Such integration would also strengthen the urban science dimension of the framework.

Fifth, the model was evaluated primarily through reconstruction metrics, classification metrics, and SHAP interpretation. Future work should further assess practical usability in real analytical workflows. This may include expert evaluation by judicial analysts, public safety researchers, or law enforcement data specialists. Such evaluation is necessary to determine whether the predictions and explanations are understandable, trustworthy, and useful for human decision support.

Despite these limitations, the findings indicate that reconstruction-aware modeling provides a useful methodological pathway for improving incomplete judicial archives. By linking structured judicial data, label-excluded heterogeneous graph temporal reconstruction, downstream charge classification, and interpretable machine learning, the proposed framework provides a useful pathway for public safety data analytics under incomplete, heterogeneous, and imbalanced legal records, while its conclusions should be interpreted within the fixed-classifier and standardized-mask evaluation scope.

## Data Availability

The judicial documents analyzed in this study were obtained from publicly accessible legal records. The processed person-level dataset is not publicly available because it contains case-level information extracted from judicial documents and may be subject to legal, privacy, and institutional restrictions. Derived and anonymized data may be made available by the corresponding author upon reasonable request, subject to institutional approval.
